# Krisanaklan Reduces Intestinal Anion and Fluid Secretion Through Inhibition of Na^+^/K^+^‐ATPase and K^+^ Channel Activity

**DOI:** 10.1111/nyas.70152

**Published:** 2025-12-16

**Authors:** Tessa A. Groeneweg, Erdene Baigal, Anny Leung, Gert‐Jan Kremers, Marcel J. C. Bijvelds

**Affiliations:** ^1^ Department of Gastroenterology and Hepatology Erasmus MC University Medical Center Rotterdam the Netherlands; ^2^ Erasmus Optical Imaging Center, Department of Pathology Erasmus MC University Medical Center Rotterdam the Netherlands

**Keywords:** cardiotonic steroids, CFTR, cholera, Na^+^/K^+^‐ATPase, oral rehydration therapy, ouabain, secretory diarrhea

## Abstract

Krisanaklan (KK) is a traditional herbal remedy used to treat an array of gastrointestinal complaints, including infectious, secretory diarrhea (SD). We assessed the effect of KK on anion and fluid secretion across intestinal epithelia, and delineated its mode of action. KK inhibited cholera toxin/cAMP‐dependent anion secretion across intestinal epithelial monolayers and native intestinal epithelium *ex vivo*. Similarly, KK reduced cAMP‐dependent fluid secretion in intestinal organoids. KK inhibited Na^+^,K^+^‐ATPase (NKA)‐mediated, ouabain‐sensitive ion transport and channel‐mediated K^+^ efflux across the basolateral plasma membrane but did not block cAMP‐dependent anion transport across the apical plasma membrane. KK also inhibited ouabain‐insensitive ATPase activity, but did not affect cAMP‐dependent protein phosphorylation. KK reduced carrier‐mediated amino acid uptake in Caco‐2 cells and Na^+^‐coupled glucose transport in porcine intestine. Further, KK inhibited cGMP‐ and Ca^2+^‐linked anion secretion across intestinal epithelial monolayers. We conclude that KK blocks intestinal epithelial anion and fluid secretion by inhibition of the NKA and K^+^ channels. Consequently, KK may ameliorate SD caused by enteric microbial pathogens. However, by inhibiting the activity of Na^+^‐dependent solute carriers, it is also predicted to counteract oral rehydration therapy, the current mainstay for SD therapy.

## Graphical abstract

Krisanaklan reduces CFTR‐dependent intestinal chloride and fluid secretion by inhibiting the Na^+^,K^+^‐ATPase and K^+^ channels in epithelial cells. Consequently, this natural, plant‐derived product may limit secretory diarrhea caused by a diverse array of microbial pathogens. However, by dissipating the transmembrane Na^+^ gradient, Krisanaklan also inhibits SGLT1‐mediated, Na^+^‐coupled glucose uptake. Therefore, it must not be used as adjunct to oral rehydration solution (ORS).

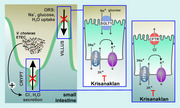

**Abbreviations**
IBMX: 3‐isobutyl‐1‐methylxanthine
PKA: cAMP‐dependent protein kinase
CTS: cardiotonic steroids
CTX: cholera toxin
CFTR: cystic fibrosis transmembrane conductance regulator
ETEC: enterotoxigenic *Escherichia coli*
P_i_
: inorganic phosphate
KK: Krisanaklan
NKCC1: Na^+^,K^+^,2Cl^−^‐cotransporter
NKA: Na^+^,K^+^‐ATPase
SD: secretory diarrhea
Isc: short‐circuit current
TEER: transepithelial electrical resistance
VIP: vasoactive intestinal peptide
VASP: vasodilator‐stimulated phosphoprotein

## 1 Introduction

Infectious diarrhea claims >1 million lives every year [1, 2]. Most cases occur in developing areas of the world, in children <5 years of age. Without adequate care, even those who manage to survive the consequences of acute dehydration remain weakened and vulnerable to reinfection, and can experience long‐lasting consequences [3]. In the Western world, deaths caused by diarrhea are rare, but with an estimated incidence of >75 million episodes annually in the United States alone, the distress and financial burden of diarrheal illness are nevertheless considerable [4].

Common pathogens that infect the intestine are *Campylobacter* spp, *Salmonella* spp, *Shigella* spp, *Yersinia* spp, pathogenic *Escherichia coli*, and rotavirus [2]. In situations of poor water and food hygiene, *Vibrio cholerae* is a major cause of epidemic diarrhea (>1 million episodes annually), and enterotoxigenic *Escherichia coli* (ETEC) account for ~70% of cases of traveler's diarrhea [2, 5]. These latter species produce toxins that, through anomalous, protracted activation of intestinal anion and fluid secretion, as well as inhibition of Na^+^ absorption and attendant osmotic fluid uptake, cause secretory diarrhea (SD) [5].

Current treatment of SD centers on replenishment of water and electrolytes to limit the extent of dehydration [1, 2]. Therapeutics that actually reduce salt and water secretion are, as yet, not routinely used in a clinical setting, but have been the focus of intense study [1, 6]. Among these, several natural products, used in some areas of the world for the management of various forms of intestinal distress, have garnered interest because they have been shown to reduce intestinal anion and/or fluid secretion in cell and animal models of SD [7, 8, 9, 10, 11, 12, 13, 14, 15, 16, 17]. Typically, these products contain (sometimes undefined) active ingredients that act by blocking: (1) the cystic fibrosis transmembrane conductance regulator (CFTR), the major, cAMP‐regulated, anion channel that mediates Cl^−^ and HCO_3_^−^ exit across the luminal (apical) plasma membrane of intestinal cells; (2) epithelial K^+^ channels, required for sustaining the membrane potential that drives anion efflux; or (3) Ca^2+^‐activated Cl^−^ channels involved in neuronal control of intestinal motility and, putatively, Cl^−^ exit from enterocytes.

One such product is Krisanaklan (KK), a mixture of extracts derived from different species of herbal plants, which is commonly used in Thailand for the treatment of gastrointestinal complaints, including stomach ache, flatulence, and abdominal distension. A previous study reported that KK ameliorates cholera toxin (CTX)‐induced SD in an animal model, and that it contains several active components, which target separate ion transport mechanisms in intestinal cells [14]. Notably, whole cell patch‐clamp experiments indicated that KK inhibits CFTR activity, as well as a Ca^2+^‐dependent Cl^−^ conductance. Interestingly, though, KK appeared to be a more potent blocker of cAMP‐dependent anion secretion across intact epithelia (achieving complete inhibition at a concentration of 0.02%) than its only moderately potent action on CFTR in cell patch‐clamp (partial inhibition at 0.05%) would suggest. Therefore, we surmised that KK may target additional elements of the cellular anion secretory machinery, and ventured to further delineate its mechanism of action.

## 2 Methods

### 2.1 Human and Porcine Intestinal Tissue, Cell Lines, and Chemicals

Rectal biopsy specimens were obtained from healthy donors (aged >18 years) who had agreed to tissue collection by giving written informed consent. The study was approved by the institutional review board of the Erasmus MC (MEC2012.233).

Intestinal tissue was obtained from crossbred Yorkshire‐Landrace pigs, aged 10–16 weeks. Animals received sufentanil (10 µg/(kg·h); iv) and were ventilated with sevoflurane (2−3%). After sternotomy, they were euthanized by inducing ventricular fibrillation with a 9 V battery. Intestinal tissue was collected and prepared for epithelial current measurements as described elsewhere [18].

The human colonic adenocarcinoma cell lines Caco‐2, HT29‐CL19A, and T84 were cultured on plastic supports essentially as described previously [19, 20, 21]. For intestinal epithelial current measurements, cells were seeded (1×10^5^ cells/cm^2^) on a permeable membrane support (Transwell #3470; Corning) that had been pretreated with collagen type I (10 µg/mL in phosphate buffered saline, 0.3 mL/cm^2^, 2 h, 37°C), and cultured for ~14 (HT29‐CL19A, T84) or 30 (Caco‐2) days.

Cell culture media and TrypLE Express were obtained from Gibco/Thermo Fisher Scientific. Further chemicals, including analytical grade inorganic salts, were purchased from Sigma‐Aldrich, except BPO‐27 (MedChemExpress.com), KK (Kilin brand, Thailand), linaclotide (Adooq Bioscience), nystatin (Santa Cruz Biotechnology), and vasoactive intestinal peptide (VIP; Bachem). KK contains ethanol (54%) and extracts from *Aquilaria crassna* (agarwood), *Syzygium aromaticum* (clove), *Terminalia triptera*, and camphor [14].

### 2.2 Organoid Culture

Organoids were cultured from human rectal biopsies or excised porcine intestinal tissue, and were maintained in medium containing the growth factors Wnt3a, Noggin, R‐Spondin 1, and EGF in Matrigel matrix (Corning) according to established protocols [22].

Culture of epithelial monolayers was performed essentially as described elsewhere [23]. In brief, extracellular matrix‐embedded organoids were suspended in advanced DMEM (4°C) and washed by centrifugation to remove the matrix. Organoids were dissociated by incubation in TrypLE Express, followed by repeated aspiration through a pipette tip. After passing through a cell strainer (70 µm; Falcon), cells were seeded (6×10^5^ cells/cm^2^) on a Transwell insert (#3470; Corning) that had been pretreated with diluted Matrigel (1:20 in phosphate‐buffered saline, 0.3 mL/cm^2^, 2 h, 37°C). Cells were cultured until a confluent monolayer was obtained (~14 days).

### 2.3 Western Blot Analysis

To assess phosphorylation of the vasodilator‐stimulated phosphoprotein (VASP), HT29‐CL19A cells were cultured in 12‐well plates until confluent, and incubated (60 min at 37°C, 5% CO_2_) with CTX (1 µg/mL) or forskolin (10 µmol/L), in the presence or absence of KK (0.1%), in Meyler solution: 128 mM NaCl, 4.7 mM KCl, 1.3 mM CaCl_2_, 1.0 mM MgCl_2_, 20 mM NaHCO_3_, 0.4 mM NaH_2_PO_4_, 0.3 mM Na_2_HPO_4_, 10 mM 4‐(2‐hydroxyethyl)‐piperazine‐1‐ethanesulfonic acid (HEPES), supplemented with glucose (10 mmol/L). Subsequently, cells were lysed in NaCl (150 mmol/L), Tris/HCl pH 7.6 (25 mmol/L), Triton X100 (1%), sodium deoxycholate (1%), sodium dodecyl sulfate (0.1%), NaF (5 mmol/L), Na_3_VO_4_ (3 mmol/L), supplemented with a protease inhibitor cocktail (Thermo Fisher Scientific). Lysates were subjected to SDS‐PAGE, and proteins were transferred to a nitrocellulose membrane. Ser‐157‐phosphorylated VASP (pVASP) was detected using antibody #3111 (1:1000; Cell Signaling Technology). β‐actin (Sc‐47778, 1:5000; Santa Cruz Biotechnology) served as a loading control. Primary antibodies were visualized using fluorescently labeled secondary antibodies (Licor Biotech) and an Odyssey imaging system (Licor Biotech). Fluorescence was quantified using Image Studio Lite software (v. 5.2; Licor Biotech).

### 2.4 Intestinal Epithelial Current Measurements

Porcine duodenum or ileum, from which the outer muscle layers were removed by blunt dissection, or epithelial monolayers cultured on Transwell inserts were inserted in Ussing chambers (P2300, Physiologic Instruments), and bathed in Meyler solution (37°C; see Section 2.3), gassed with 95% O_2_, 5% CO_2_. In assays on porcine tissue, the cyclooxygenase inhibitor indomethacin (10 µmol/L) was added to the Meyler solution to reduce endogenous production of prostaglandins. Glucose (10 mmol/L) was added to the contraluminal (basolateral) bathing solution in assays on porcine tissue, but added to both compartments in all other instances. The transepithelial potential difference was clamped at 0 mV using a VVC‐MC8 module (Physiologic Instruments), and the resulting short‐circuit current (Isc) was recorded using an analog‐to‐digital signal converter (sample frequency: 1 Hz; PowerLab 8/35, AD Instruments) and LabChart 8 software (AD Instruments). Where indicated, nystatin (0.4 mg/mL) was added to the luminal or basolateral bathing solution to render the plasma membrane permeable to monovalent ions. In some experiments where nystatin was applied, part of the NaCl in the contraluminal bathing solution was isosmotically replaced by sodium‐isethionate, lowering the Cl^−^ concentration of the solution to 46 mmol/L. In other experiments, part of the NaCl in the luminal bathing solution was substituted by KCl, thus setting the K^+^ concentration at 47 mmol/L. Osmolality of bathing solutions was routinely checked using a cryoscopic osmometer (Osmomat 30, Gonotec GmbH), and ranged between 294 and 306 mOsm/kg.

CFTR‐dependent Isc responses in tissue and monolayers were triggered by the addition of the adenylyl cyclase activator forskolin (10 µmol/L), CTX (1 µg/mL), the combination of VIP (50 nmol/L; added to the basolateral compartment) and the broad‐specificity phosphodiesterase inhibitor isobutyl‐methylxanthine (IBMX; 50 µmol/L), linaclotide (1 µmol/L; added luminally), or carbachol (200 µmol/L; added basolaterally). KK (concentration range: 1×10^−4^−2×10^−1^ %) and the CFTR inhibitor BPO‐27 (20 µmol/L) were added to both the luminal and basolateral compartments. Ouabain (50 µmol/L) and BaCl_2_ (1 mmol/L) were used to inhibit Na^+^,K^+^‐ATPase (NKA), and K^+^ channel activity, respectively, and added basolaterally.

### 2.5 Cellular ATPase Activity

ATPase activity in HT29‐Cl19A homogenates was assessed using a colorimetric assay that detects inorganic phosphate (P_i_) production essentially as described in detail elsewhere [24]. In brief, HT29‐Cl19A cells were suspended in sucrose (250 mmol/L; ~3×10^6^ cells/mL) and homogenized using a Potter‐Elvehjem type homogenizer. After the protein concentration was determined using a reagent kit (Bio‐Rad), homogenates were snap‐frozen in liquid N_2_ and stored at ‐80°C until further use. After thawing, homogenates were treated with saponin (0.2 mg/mL) and diluted (1:20) in: 100 mM NaCl, 15 mM KCl, 5 mM MgCl_2_, 3 mM ATP, 0.1 mM EDTA, 30 mM imidazole, set at pH 7.4 with HEPES, supplemented with KK (concentration range: 0.01–1%). To assess NKA‐independent P_i_ production, some assays were performed in medium without KCl, to which ouabain (1 mmol/L) was added. Assay mixtures were incubated for 60 min at 37°C, and reactions were quenched by adding trichloroacetic acid (5%). After adding one volume of color reagent (0.32 mol/L FeSO_4_, 9 mmol/L (NH_4_)_6_Mo_7_O_24_ in 3.5% H_2_SO_4_), P_i_ production was quantified by measuring absorbance at 700 nm (Infinite M Nano plate reader, Tecan). A NaH_2_PO_4_ solution (10 mmol/L) served as a standard.

### 2.6 Fluid Transport in Organoids

CFTR‐mediated anion secretion stimulates osmotic fluid transport into the enclosed lumen of intestinal organoids, leading to organoid swelling [22]. To evaluate the effect of KK on fluid secretion, porcine colonic organoids were seeded in 12‐well cell culture plates in droplets (5 µL) of extracellular matrix (Matrigel). After organoids were maintained for 24 h at 37°C in 5% CO_2_, CFTR‐dependent organoid swelling was triggered by the addition of forskolin (5 µmol/L) in the presence or absence of KK (0.1%). To evaluate organoid swelling, the total area occupied by organoids in each droplet (containing ~30 organoids) was quantified at the start of the experiment and after incubation with forskolin (6 h). To this end, embedded organoids were visualized by bright‐field microscopy on an EVOS FL cell imaging system (2× objective; Invitrogen). Micrographs were analyzed on the ImageJ platform (https://imagej.net) using a software module developed by the Optical Imaging Center of the Erasmus MC that fully automates image analysis.

### 2.7 Data Analysis

Data are presented as mean ± SD. Statistical analysis was performed as indicated in the figure legends by Student's *t*‐test or, when multiple groups were compared, repeated measures one‐way ANOVA, using the method of Sidak to correct for multiple comparisons (SPSS v. 28, IBM).

## 3 Results

### 3.1 KK Inhibits cAMP‐Dependent Anion Secretion in Intestinal Epithelial Monolayers and Intestinal Tissue

**FIGURE 1:**
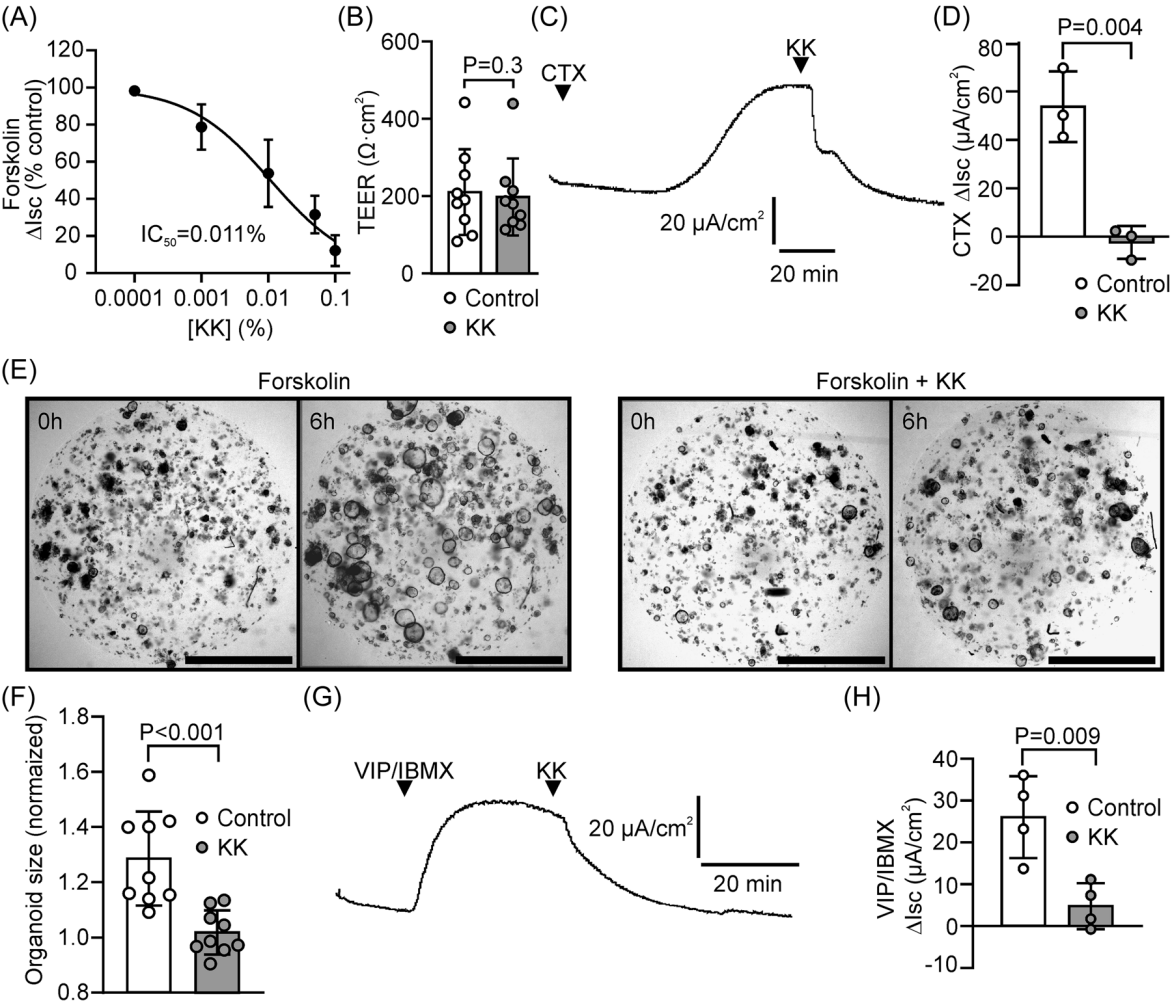
Krisanaklan (KK) inhibits intestinal epithelial anion and fluid secretion. (A) Concentration‐dependence of the effect of KK on the forskolin‐dependent Isc response assessed in HT29‐CL19A epithelial monolayers (mean ± SD). For deriving the half maximal inhibitory concentration (IC_50_), data of between 3 and 9 technical replicates per data point were analyzed by nonlinear regression. (B) The transepithelial electrical resistance (TEER; mean ± SD) of HT29‐CL19A epithelial monolayers in the presence or absence of KK (0.1%). Each data point represents one technical replicate. Statistical analysis: paired *t*‐test. (C) Representative experiment showing the effect of KK (0.1%) on the cholera toxin (CTX)‐dependent Isc response assessed in epithelial monolayers cultured from human intestinal organoids. (D) Aggregate data of CTX‐dependent Isc response in organoid‐derived epithelial monolayers in the presence or absence of KK (mean ± SD). Each data point represents one technical replicate. Statistical analysis: *t*‐test. (E) Bright‐field images of porcine colonic organoids embedded in extracellular matrix domes just before (0 h) and after 6 h of forskolin treatment, in the presence (right panel) or absence (left) of KK (0.1%). Scale bar: 500 µm. (F) Aggregate data of forskolin‐dependent organoid swelling. Data depict organoid size after 6 h forskolin treatment, relative to the size at the start of the experiment (mean ± SD). Each data point represents one technical replicate. Statistical analysis: *t*‐test. (G) The effect of KK (0.1%) on the vasoactive intestinal peptide (VIP)‐ and isobutyl‐methylxanthine (IBMX)‐dependent Isc response of pig ileum, *ex vivo*. (H) Aggregate data of the VIP/IBMX‐dependent Isc responses in pig ileum in the presence or absence of KK (mean ± SD). Each data point represents one biological replicate. Statistical analysis: *t*‐test.

Epithelial monolayers of the intestinal cell line HT29‐CL19A were mounted in Ussing chambers, and used for assessing electrogenic ion transport. Addition of the adenylyl cyclase agonist forskolin, which induces cAMP‐dependent phosphorylation/activation of CFTR, increased the Isc by 58 ± 12 µA/cm^2^ (*n* = 9). KK concentration‐dependently inhibited the forskolin‐dependent Isc response, with a half maximal inhibitory concentration (IC_50_) of 0.011% (95% CI: 0.007−0.015%; Figure 1A). The transepithelial electrical resistance of the monolayers was not affected by KK treatment (Figure 1B).

CTX, through covalent modification of the G_α,s_‐subunit of G‐protein‐coupled receptors, provokes sustained adenylyl cyclase activation and cAMP production in intestinal cells [5]. Treatment of rectal organoid‐derived intestinal epithelial monolayers with CTX, which enters the cells through endocytosis and undergoes retrograde trafficking to the endoplasmic reticulum before reaching its target, led to a gradual increase in the Isc (Figure 1C). Addition of KK (0.1%) promptly reversed the effect of CTX on the Isc (Figure 1C,D).

Forskolin‐dependent intestinal organoid swelling, as a result of osmotic fluid transport, is fully dependent on CFTR [22]. When porcine colonic organoids were treated with KK, forskolin‐dependent fluid accumulation was significantly attenuated (Figure 1E,F). These data demonstrate that KK effectively inhibits CFTR‐dependent anion and fluid secretion.

Many inhibitors of CFTR‐dependent anion and fluid secretion, although highly effective in vitro, in cell models, have shown limited efficacy in intestinal tissue, ex vivo, let alone in clinical trials [25, 26, 27]. To assess the effect of KK on the anion secretory response of native tissue, we used porcine intestinal tissue, whose dimensions (villus length, crypt depth, thickness of the mucosa) approximate those of the human intestine. Small intestinal tissue sheets were mounted in Ussing chambers, and the combination of VIP and the phosphodiesterase inhibitor IBMX was applied to elicit cAMP‐dependent anion secretion. KK (0.1%) acutely lowered the Isc response induced by combined VIP/IBMX administration, demonstrating that it blocks anion secretion in intestinal tissue (Figure 1G,H).

### 3.2 KK Does Not Inhibit PKA‐Dependent Protein Phosphorylation

**FIGURE 2:**
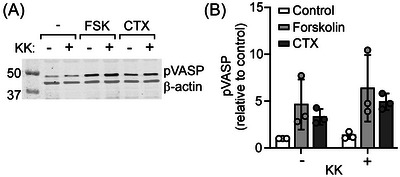
Krisanaklan (KK) does not inhibit cAMP‐dependent protein phosphorylation. HT29‐CL19A cells were incubated with either forskolin (FSK) or cholera toxin (CTX) in the presence or absence of KK (0.1%), and phosphorylation of VASP was assessed by western blot analysis. (A) Representative western blot. Outer left lane shows markers with molecular weight (kDa) as indicated. β‐actin served as loading control. (B) Effect of KK on phosphorylated VASP levels in forskolin‐ or CTX‐treated cells (mean ± SD). Each data point represents one technical replicate.

Activation of CFTR upon stimulation of intestinal cells by forskolin, CTX, or VIP is critically dependent on the activity of the cAMP‐dependent protein kinase (PKA) [5]. Therefore, we considered that KK may affect PKA‐dependent activation of CFTR by lowering cAMP production or by directly inhibiting the activity of the PKA catalytic subunit. We assessed cAMP‐dependent phosphorylation of VASP, a protein that, like CFTR, is located at the apical pole of intestinal epithelial cells, to monitor the effect of KK on PKA activity. We observed that both forskolin and CTX increased phosphorylated VASP levels in HT29‐CL19A cells, consistent with their stimulatory effect on CFTR‐dependent anion secretion. However, although KK markedly attenuated the prosecretory effects of both forskolin and CTX in intestinal cells, it did not prevent forskolin‐ or CTX‐dependent phosphorylation of VASP (Figure 2). These data indicate that KK does not inhibit epithelial anion secretion by impairing PKA function and CFTR phosphorylation/activation.

### 3.3 KK Blocks Epithelial Anion Secretion, But Fails to Block Cl^−^ Transport Across the Apical Plasma Membrane of Intestinal Cells

**FIGURE 3:**
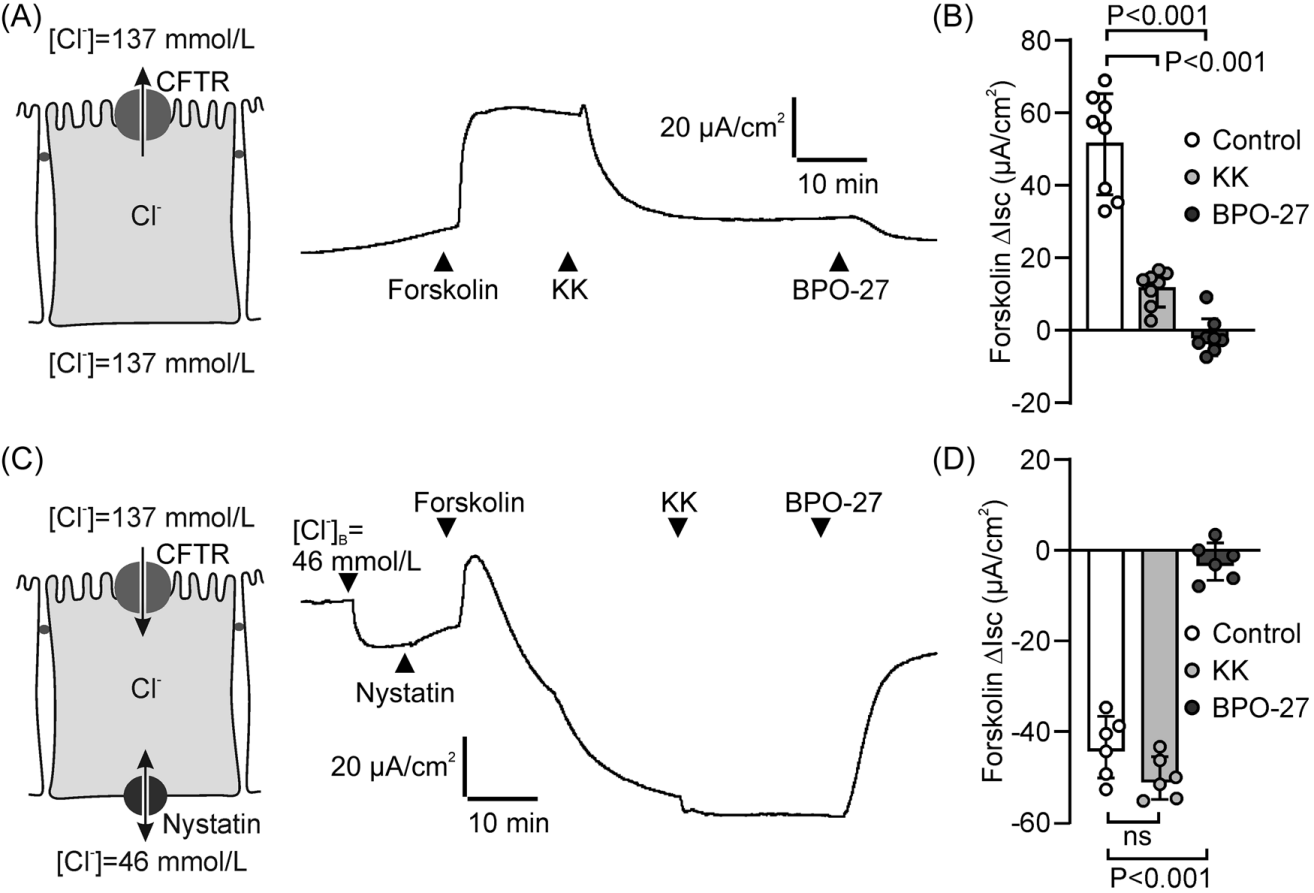
The CFTR blocker BPO‐27, but not Krisanaklan (KK), inhibits forskolin‐dependent anion transport across intestinal epithelial monolayers in which the basolateral plasma membrane is rendered permeable to monovalent ions. (A) Representative experiment showing the effect of KK (0.1%) and BPO‐27 (20 µmol/L) on forskolin‐dependent anion transport across intact HT29‐CL19A epithelial monolayers. (B) Aggregate data of the forskolin‐dependent Isc response in the presence or absence of KK or BPO‐27 (mean ± SD). Each data point represents one technical replicate. Statistical analysis: ANOVA. (C) Representative experiment showing the effect of KK (0.1%) and BPO‐27 (20 µmol/L) on forskolin‐dependent anion transport across nystatin‐treated HT29‐CL19A epithelial monolayers in the presence of an apical‐to‐basolateral chloride gradient. (D) Aggregate data of the forskolin‐dependent Isc response in permeabilized HT29‐CL19A epithelial monolayers in the presence or absence of KK or BPO‐27 (mean ± SD). Each data point represents one technical replicate. Statistical analysis: ANOVA.

We asked whether KK inhibits epithelial anion secretion by directly blocking CFTR function. First, we verified that, in HT29‐CL19A monolayers, KK effectively inhibited the forskolin‐dependent Isc response, and that this secretory response was fully blocked by the CFTR inhibitor BPO‐27 (Figure 3A,B). Next, to assess whether KK directly targets CFTR, we investigated forskolin‐dependent Cl^−^ transport across monolayers in which the basolateral plasma membrane was made permeable to monovalent ions by nystatin treatment. As in intact monolayers, forskolin readily induced an Isc response, consistent with activation of CFTR (note that because of the imposed apical‐to‐basolateral Cl^−^ gradient, the direction of the Isc response is reversed, compared to nonpermeabilized cells). Strikingly, KK failed to attenuate the Isc response under these conditions, whereas the CFTR blocker BPO‐27 effectively blocked the response, similar as in nonpermeabilized cells (Figure 3C,D). These results indicated that KK targets (an) element(s) of the anion secretory mechanism operating upstream of CFTR, that is/are required for maintaining the electrochemical potential that drives CFTR‐mediated anion efflux.

### 3.4 KK Inhibits the Na^+^,K^+^‐ATPase

We hypothesized that KK may target a basolateral ion transporter involved in transepithelial Cl^−^ secretion. Cellular Cl^−^ uptake across the basolateral plasma membrane is mediated by the Na^+^,K^+^,2Cl^−^ cotransporter NKCC1 (*SLC12A2*). NKCC1‐mediated Cl^−^ uptake is driven by an inwardly directed electrochemical Na^+^ gradient, which is maintained by the NKA, also located in the basolateral plasma membrane. Transepithelial Cl^−^ secretion is predicted to be strictly dependent on the activity of this cation pump [5].

**FIGURE 4:**
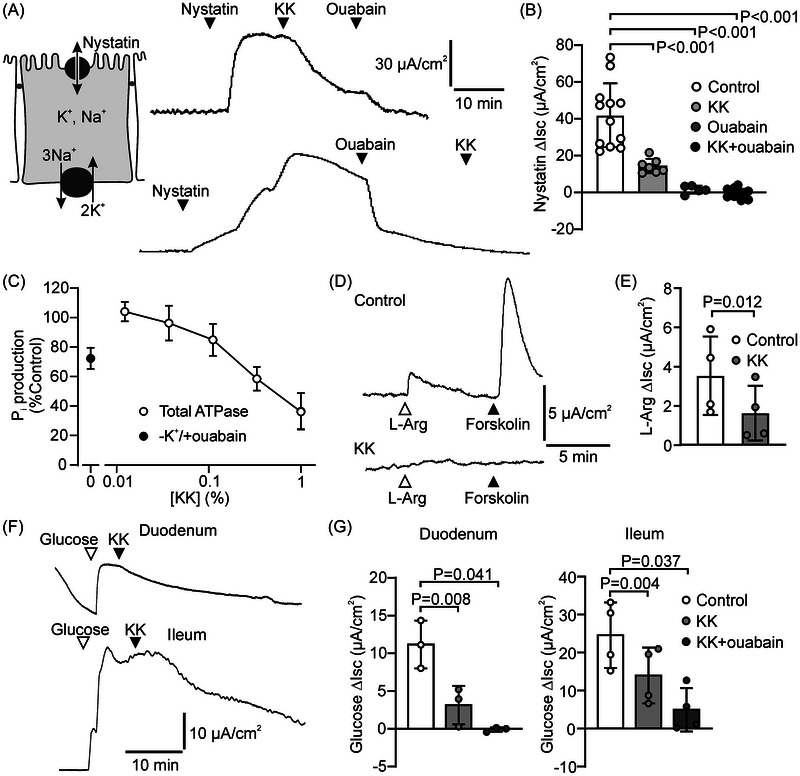
Krisanaklan (KK) inhibits the Na^+^,K^+^‐ATPase (NKA) and secondary active solute transport in intestinal epithelia. (A) Representative experiment showing the effect of KK (0.1%) and the NKA blocker ouabain (50 µmol/L) on ion transport across HT29‐CL19A epithelial monolayers in which the apical plasma membrane was permeabilized with nystatin. (B) Aggregate data of the nystatin‐dependent Isc response of HT29‐Cl19A monolayers in the presence or absence of KK and/or ouabain (mean ± SD). Each data point represents one technical replicate. Statistical analysis: ANOVA. (C) Concentration‐dependence of the effect of KK on inorganic phosphate (P_i_) production in HT29‐CL19A cell homogenates (mean ± SD). For comparison, NKA‐independent P_i_ production was assessed in medium containing ouabain but no K^+^. (D) Representative experiment showing the effect of KK (0.2%) on the l‐arginine (l‐Arg; 10 mmol/L)‐ and forskolin‐dependent Isc response of Caco‐2 epithelial monolayers. (E) Aggregate data of the l‐Arg‐dependent Isc response in the presence or absence of KK. Each data point represents one technical replicate (mean ± SD). Statistical analysis: *t*‐test. (F) Representative experiment showing the effect of KK (0.2%) on the luminal glucose (10 mmol/L)‐dependent Isc response of porcine duodenum and ileum, ex vivo. (G) Aggregate data of the glucose‐dependent Isc response in porcine small intestine, in the presence or absence of KK and ouabain. Each data point represents one technical replicate (mean ± SD). Statistical analysis: ANOVA.

To assess the effect of KK on NKA activity, we used nystatin to permeabilize the apical plasma membrane of HT29‐CL19A epithelial monolayers. Nystatin treatment induced a marked Isc response, consistent with the 3Na^+^:2K^+^ stoichiometry of the ATPase (Figure 4A,B). Because no transepithelial ion gradients were imposed in these experiments (i.e., unmodified Meyler was used in both compartments), any contribution of ion channels to this response can be excluded. Indeed, ouabain blocked the nystatin‐dependent Isc response completely, indicating that NKA activity fully accounts for this current. Importantly, we found that KK mimicked the effect of ouabain, albeit that it did not fully block the response (Figure 4B).

Next, we used HT29‐Cl19A cell homogenates to assess the effect of KK on cellular ATPase activity, by measuring P_i_ production in the presence of ATP. The NKA accounts for a substantial part of this production: addition of ouabain, combined with the omission of K^+^ from the reaction mixture, led to a ~30% reduction in P_i_ production (Figure 4C). We found that KK concentration‐dependently reduced P_i_ production, consistent with its effect on the NKA‐mediated Isc response of permeabilized monolayers. Interestingly, KK, at concentrations ≥0.3% reduced P_i_ production more than ouabain addition/K^+^ omission, suggesting that KK also affects other ATPases and/or P_i_‐producing processes.

Considering its effect on NKA activity, KK may not only reduce NKCC1 activity, but also affect other secondary active (Na^+^‐dependent) transport mechanisms in intestinal cells. The small intestine expresses an array of Na^+^‐ or H^+^‐coupled transport proteins that are involved in the absorption of amino acids, and glucose is absorbed through the Na^+^‐glucose cotransporter SGLT1 (*SLC5A1*) [5, 28]. In Caco‐2 monolayers, we observed that the addition of l‐arginine to the luminal bathing solution increased the Isc, consistent with carrier‐mediated amino acid uptake (Figure 4D) [20, 29]. KK significantly attenuated the response of Caco‐2 monolayers to l‐arginine (Figure 4E). In porcine duodenum and ileum, the Isc response elicited by the addition of glucose (10 mmol/L) to the luminal bath was attenuated by KK, as well as by ouabain (Figure 4F,G). These data suggest that KK, by inhibition of the NKA, impedes the activity of secondary active solute carriers.

### 3.5 KK Inhibits K^+^ Channel Activity

**FIGURE 5:**
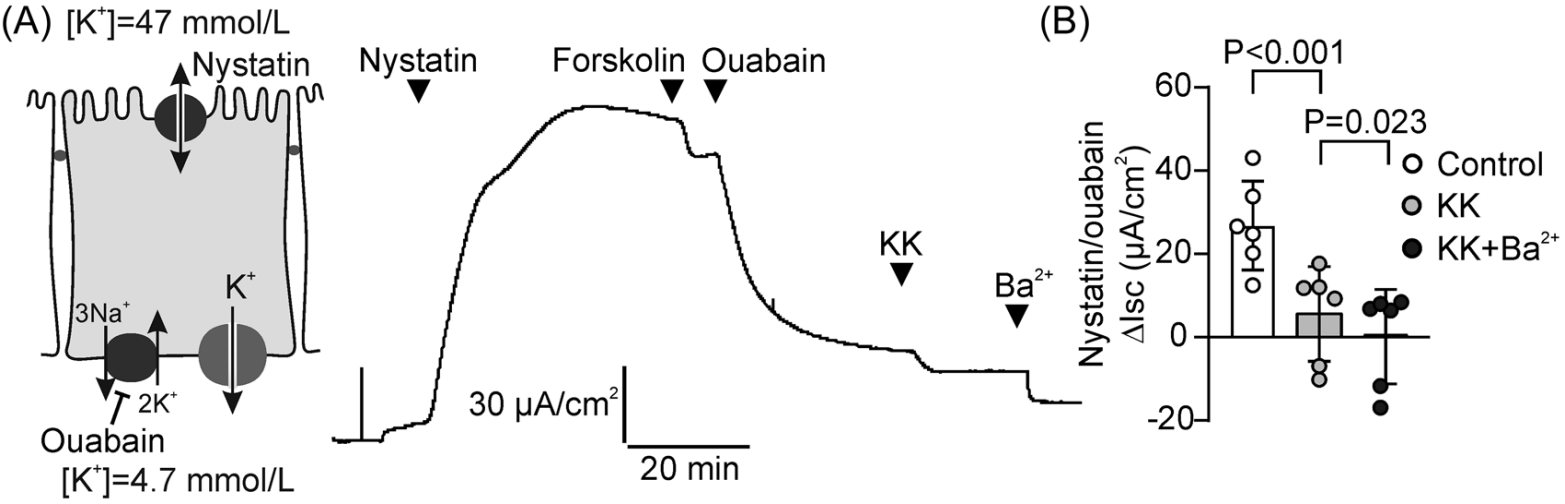
Krisanaklan (KK) inhibits epithelial K^+^‐channels. (A) Representative experiment showing the effect of KK (0.1%) and the K^+^‐channel blocker Ba^2+^ (1 mmol/L) on ion transport across HT29‐CL19A epithelial monolayers in which the apical plasma membrane was rendered permeable to monovalent ions by nystatin treatment. Ouabain (50 µmol/L) was used to block NKA‐dependent ion transport, and Isc responses were assessed in the presence of an apical‐to‐basolateral K^+^ gradient. (B) Aggregate data of the nystatin‐dependent, ouabain‐insensitive Isc response of permeabilized HT29‐Cl19A monolayers in the presence or absence of KK or the combination of KK and Ba^2+^ (mean ± SD). Each data point represents one technical replicate. Statistical analysis: ANOVA.

Both NKCC1 and NKA activity, and consequently Cl^−^ secretion, depend on the opening of channels that allow cellular efflux (recycling) of accumulating K^+^ [5, 30]. Therefore, we speculated that KK may also reduce anion secretion through inhibition of K^+^ efflux. To assess the effect of KK on K^+^ channel activity, we used nystatin to permeabilize the apical plasma membrane of HT29‐CL19A epithelial monolayers, and added ouabain to negate the contribution of the NKA to the nystatin‐dependent Isc. Further, we increased the K^+^ concentration in the luminal bath to impose an apical‐to‐serosal gradient, and added forskolin to activate cAMP‐dependent K^+^‐channels [30]. Because combined ouabain/nystatin treatment increases the intracellular Ca^2+^ concentration, this protocol is also predicted to activate Ca^2+^‐dependent K^+^ channels [31]. Under these conditions, we found that KK significantly reduced the Isc, indicating that it inhibits cellular K^+^ secretion (Figure 5). Addition of the broad‐specificity K^+^ channel blocker Ba^2+^ led to a further reduction in the Isc, and completely abolished the nystatin‐dependent, ouabain‐insensitive, Isc response.

### 3.6 KK Inhibits cGMP‐ and Ca^2+^‐Dependent Anion Secretion in Intestinal Epithelial Monolayers

**FIGURE 6:**
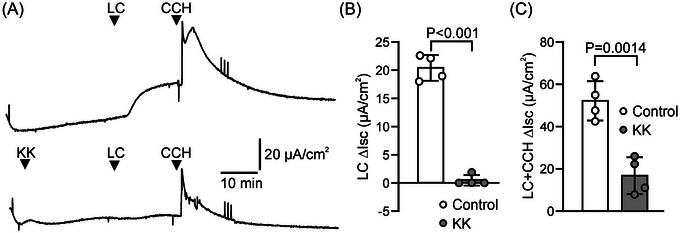
Krisanaklan (KK) inhibits cGMP‐ and Ca^2+^‐dependent anion secretion. (A) Representative experiment showing the effect of linaclotide (LC; 1 µmol/L) and carbachol (CCH; 200 µmol/L) on anion secretion across T84 epithelial monolayers, in the presence (bottom panel) or absence (upper panel) of KK (0.1%). (B) Aggregate data of the LC‐dependent Isc response in the presence or absence of KK (mean ± SD). Each data point represents one technical replicate. Statistical analysis: *t*‐test. (C) Aggregate data of the combined LC/CCH‐dependent Isc response in the presence or absence of KK (mean ± SD). Because the Isc response to CCH was highly transient, the mean of the Isc response recorded over the 180 s interval immediately after CCH addition is presented. Each data point represents one technical replicate. Statistical analysis: *t*‐test.

From the observation that KK inhibited both NKA and K^+^‐channel activity, we inferred that KK may also inhibit cGMP‐ and Ca^2+^‐linked anion secretion. To test this assumption, we assessed the effect of KK on the anion secretory response of T84 cells to linaclotide, a synthetic analog of the heat‐stable toxin (STa) produced by ETEC. The guanylyl cyclase C ligand STa was previously shown to increase cGMP in T84 cells and trigger opening of K^+^ and Cl^−^ channels [21]. We observed that in T84 monolayers, KK strongly inhibited the anion secretory response to linaclotide (Figure 6A,B).

In the sustained presence of linaclotide, the muscarinic receptor agonist carbachol transiently enhanced the secretory response of T84 monolayers, consistent with the presence of G_q_‐coupled M_3_ muscarinic receptors on intestinal epithelial cells. Principally, M_3_ stimulation is thought to enhance CFTR‐mediated intestinal anion secretion through activation of Ca^2+^‐dependent K^+^ channels, which leads to cell membrane hyperpolarization [5]. The response to carbachol was markedly attenuated by KK (Figure 6A,C). These results indicate that KK may reduce the anion and fluid secretory response to both cGMP‐linked enterotoxins and (endogenous) Ca^2+^ agonists.

## 4 Discussion

In this study, we demonstrate that KK, a natural herbal remedy used for the treatment of gastrointestinal complaints, inhibited cAMP‐dependent transepithelial anion and fluid secretion in intestinal cells, in vitro, as well as in native intestinal tissue. KK exerts this antisecretory effect by inhibition of the NKA. In addition, it also blocks a K^+^ conductive pathway in intestinal cells. Both these actions are predicted to reduce intestinal anion and fluid secretion by decreasing the driving force for apical anion efflux. The effect of KK on NKA activity and K^+^ efflux fully accounts for its antisecretory action. We conclude that KK may effectively reduce fluid loss caused by microbial toxins and limit dehydration. However, by inhibiting the activity of Na^+^‐coupled solute carriers in intestinal cells, which includes the glucose transporter SGLT1, it will also counteract oral rehydration therapy, the current mainstay for management of SDs [1]. Therefore, these different therapeutic strategies should not be applied simultaneously.

Our data demonstrate that KK inhibits the ouabain‐sensitive NKA in intestinal epithelial cells. This Na^+^‐pump/enzyme creates the transmembrane electrochemical Na^+^ gradient that drives secondary active uptake of both Cl^−^ and HCO_3_^−^ across the basolateral plasma membrane and allows passive, channel (CFTR)‐mediated efflux of these anions across the apical plasma membrane. A diverse group of plant species produce inhibitors of the NKA. Although now largely replaced by other drugs, these so‐called cardiotonic steroids (CTS), including ouabain, have been used for many decades to treat congestive heart failure and arrhythmia [32]. By blocking the NKA, CTS raise the Ca^2+^ concentration in the cytoplasm and sarcoplasmic reticulum of cardiomyocytes, leading to an increase in contractile force and cardiac output [32]. Because KK is composed of several herbal plant extracts, it is well conceivable that it contains a natural ingredient with a CTS‐like chemical structure and biological activity. Previously, it was shown that another natural remedy, Uzara, contains CTS‐like compounds, and its antidiarrheal activity was, similarly, attributed to inhibition of the NKA [33]. Notwithstanding their activity toward such a vital and ubiquitous ion transport mechanism, Uzara and KK are generally considered safe for oral use, implying that the systemic availability of the CTS‐mimetic component in these products must be low, precluding significant adverse effects on the cardiovascular system [34].

In our assays on intestinal cell homogenates, KK lowered P_i_ production more than ouabain, indicating that KK also contains a component that inhibits ATPases other than the NKA. Consequently, it is conceivable that KK contains a pharmacophore that interacts with a critical domain shared by the structurally related (P‐type) ATPases (e.g., the ATP binding pocket). However, it is notable that CTS (including ouabain) do not, but bind to an extracellular domain distinctive of the NKA [32]. Therefore, we consider it more plausible that, apart from a component with a true CTS‐like mode of action, KK contains one or more additional component(s) that inhibit(s) the activity of other P_i_‐producing enzymes. Nevertheless, we cannot entirely exclude the possibility that KK contains a component that, unlike typical CTS, confers activity toward a wider group of ATP‐converting enzymes, including the NKA.

Independent of its effect on the NKA, KK blocked a K^+^ conductive pathway in permeabilized epithelial monolayers. K^+^ channel activity is required to maintain the electromotive force for anion extrusion by the intestinal epithelium. Apart from the principal cAMP‐ and Ca^2+^‐activated channels (KCNE3‐KCNQ1 and KCNN4, respectively), intestinal epithelial anion secretion is thought to involve several other distinct K^+^ channels, some of which have not been identified at the molecular level [30, 31, 35, 36, 37, 38]. Important for the pathophysiology of SD, by stimulating cellular K^+^ efflux, endogenous Ca^2+^ agonists (e.g., acetylcholine released by parasympathetic neurons) will strengthen the secretory response elicited by cAMP‐ or cGMP‐linked pathogenic stimuli that trigger CFTR phosphorylation/activation (e.g., CTX, STa) [5]. Therefore, inhibiting either cAMP‐ or Ca^2+^‐activated K^+^ channels, or any other K^+^ channel operative under these circumstances, may augment the antisecretory action of KK. Consistent with this scenario, we observed that KK not only inhibited the Isc response to the STa‐mimetic compound linaclotide, but also markedly attenuated the anion secretory response elicited by the muscarinic receptor agonist carbachol in the sustained presence of linaclotide.

Our data indicate that KK did not affect PKA‐mediated protein phosphorylation, which is required for activation of CFTR. Consistent with these results, KK did not affect the forskolin‐dependent, BPO‐27–sensitive apical Cl^−^ conductance in permeabilized monolayers. These data show that, while KK inhibits CFTR‐dependent anion secretion in intestinal epithelia, it does not block CFTR directly or prevent its phosphorylation/activation. Different from our present observations in intestinal epithelia, patch clamp experiments on CFTR‐expressing Fischer rat thyroid cells showed that KK partially blocked a current with CFTR‐like properties [14]. An intracellular site of action was proposed, but it is unclear whether the active component of KK entered the impaled cell from the extracellular bath, or if KK was added to the pipette solution. We infer that in our experiments, in which KK was added only to the bathing solution, this component with putative CFTR‐inhibitory properties does not partition intracellularly, and, consequently, does not contribute to the antisecretory action of KK in intestinal cells.

In this context, it is of interest that KK effectively blocked anion secretion across porcine intestinal tissue. This contrasts with data on experimental antidiarrheal drugs that specifically target CFTR, which are generally much less effective and potent in native tissue, both ex vivo and in vivo, than experiments on cell models would predict [6, 26, 27]. Primarily, this discrepancy may derive from the distinctive architecture of the intestinal mucosa. It has been put forward that compounds diffuse poorly into the mucus‐covered deeper regions of the crypts of Lieberkühn, in which the bulk of CFTR‐dependent fluid secretion occurs [25]. In particular, when CFTR is active, the fluid flow arising from the lower crypt regions is thought to effectively oppose the penetration of inhibitors [25]. In view of these considerations, the efficacy of KK observed at present in intestinal tissue reinforces the notion that the antidiarrheal action of KK in native tissue relies chiefly on the inhibition of ion transport mechanisms other than CFTR, that is, the NKA and K^+^ channels, situated in the basolateral plasma membrane of the intestinal cells. How the active component of KK passes the intestinal epithelium to reach its target is speculative. Notably, some CTS (e.g., digitoxin) are lipophilic and may enter the epithelial cells passively before binding to the NKA at the basolateral pole of the cell. Others are more hydrophilic and unlikely to enter cells via simple diffusion [32]. Notwithstanding, even luminal application of hydrophilic CTS (like ouabain) leads to inhibition of intestinal sodium and glucose absorption, in vivo, indicating that such water‐soluble CTS are absorbed either via a paracellular route, and/or via an as yet unidentified cellular mechanism [39].

Because KK is made up of extracts from multiple plant species, it consists of a highly complex mixture of organic compounds. An important limitation of our study is that it does not identify the individual components that confer activity toward the NKA and epithelial K^+^ channels. Related to this, because KK is a natural product, our study does not account for potential variation in the composition of KK between different product batches, which may affect its efficacy. Finally, we cannot exclude the possibility that KK contains yet other ingredients that contribute to its antidiarrheal properties, via (a) mechanism(s) not evaluated presently, for example, modulation of gut motility.

In conclusion, our data demonstrate that KK reduces intestinal anion and fluid secretion by inhibiting NKA and K^+^ channel activity. Consequently, KK may limit fluid loss and dehydration caused by a diverse array of microbial toxins that trigger cAMP‐, cGMP‐, or Ca^2+^‐dependent anion secretory responses in intestinal epithelium, including *V. cholerae* CTX, *E. coli* heat‐stable and ‐labile toxins, and rotavirus nonstructural protein 4 (NSP4) [5]. However, through inhibition of the NKA, KK will also dissipate the transmembrane electrochemical Na^+^ gradient that drives the activity of an array of solute carriers involved in nutrient uptake. As a result, KK is predicted to reduce the efficacy of oral rehydration therapy, which critically relies on Na^+^ and glucose uptake via one of such carriers, SGLT1. Therefore, we recommend that, while KK may be beneficial in people suffering from SD, it must not be used as an adjunct to oral rehydration solution.

## Author Contributions

**Tessa A. Groeneweg**: Investigation, formal analysis, methodology, resources, validation, writing—review and editing. **Erdene Baigal**: Investigation, validation, writing—review and editing. **Anny Leung**: Investigation, validation, writing—review and editing. **Gert‐Jan Kremers**: Methodology, resources, writing—review and editing. **Marcel J. C. Bijvelds**: Conceptualization, investigation, formal analysis, methodology, project administration, supervision, validation, visualization, writing—original draft, writing—review and editing. All authors have read and agreed to the published version of the manuscript.

## Funding

This study was supported by the Erasmus MC University Medical Center pilot grant FB386063.

## Conflicts of Interest

The authors declare that they have no known competing financial interests or personal relationships that could have appeared to influence the work reported in this paper.

## Ethics Approval Statement

The study was approved by the independent committee on ethical use of experimental animals, Rotterdam, according to national guidelines and in accordance with the Basel declaration on experimental research on vertebrates (AVD1010020173286).

## Institutional Review Board Statement

The study was conducted according to the guidelines of the Declaration of Helsinki and approved by the Institutional Review Board (Ethics Committee) of the Erasmus MC (MEC2012.233).

## Data Availability

Analytic methods and study materials will be made available to other researchers on request to the last author.
